# Supplementary material for: Burst muscle performance predicts the speed, acceleration, and turning performance of Anna’s hummingbirds
Source: eLife. 2015 Nov 19;4:e11159. doi: 10.7554/eLife.11159 (PMC4737652; doi:10.7554/eLife.11159)
Supplement: Supplementary file 1. — DOI: http://dx.doi.org/10.7554/eLife.11159.018 [file elife-11159-supp1.docx]

**Supplementary file 1**

**1A.** Candidate models of maneuvering performance metrics (n = 20 birds in 20 solo trials and 16 paired competition trials). Models are ranked by AICc, with supported models within 2 AICc units of the best-fit model highlighted in bold. Akaike weight is a measure of the probability that a given model is the best-fit model relative to others in that model set.

| model set | rank | fixed effects | df | AICc | delta AICc | Akaike weight |
| --- | --- | --- | --- | --- | --- | --- |
| *Vel_max_* | 1  2  3  4  5  6  7  8 | **competition + mass + burst + experiment + days**  **competition + mass + burst + wing aspect ratio + experiment + days**  **competition + mass + burst + wing length + experiment + days**  competition + mass + burst + wing length + wing aspect ratio + experiment + days  competition + mass + wing aspect ratio + experiment + days  competition + mass + wing length + experiment + days  competition + mass + wing length + wing aspect ratio + experiment + days  no fixed effects | 9  10  10  11  9  9  10  3 | 16.49  17.78  17.90  19.21  19.38  20.37  21.28  51.63 | 0  1.29  1.40  2.71  2.88  3.88  4.79  35.14 | 0.36  0.19  0.18  0.09  0.09  0.05  0.03  0 |
| *AccHor_max_* | 1  2  3  4  5  6  7  8 | **competition + mass + burst + experiment + days**  competition + mass + burst + wing length + experiment + days  competition + mass + burst + wing aspect ratio + experiment + days  competition + mass + wing length + experiment + days  competition + mass + wing aspect ratio + experiment + days  competition + mass + burst + wing length + wing aspect ratio + experiment + days  competition + mass + wing length + wing aspect ratio + experiment + days  no fixed effects | 9  10  10  9  9  11  10  3 | 138.72  141.60  141.76  143.30  143.40  144.80  146.25  172.31 | 0  2.88  3.04  4.58  4.68  6.07  7.53  33.59 | 0.58  0.14  0.13  0.06  0.06  0.03  0.01  0 |
| *DecHor_max_* | 1  2  3  4  5  6  7  8 | **competition + mass + burst + experiment + days**  competition + mass + burst + wing aspect ratio + experiment + days  competition + mass + burst + wing length + experiment + days  competition + mass + burst + wing length + wing aspect ratio + experiment + days  competition + mass + wing length + experiment + days  competition + mass + wing aspect ratio + experiment + days  competition + mass + wing length + wing aspect ratio + experiment + days  no fixed effects | 9  10  10  11  9  9  10  3 | 125.78  128.81  128.83  132.02  132.09  132.15  135.17  165.65 | 0  3.03  3.05  6.24  6.31  6.37  9.39  39.87 | 0.64  0.14  0.14  0.03  0.03  0.03  0.01  0 |
| *AccVU_max_* | 1  2  3  4  5  6  7  8 | **no fixed effects**  competition + mass + wing length + experiment  competition + mass + burst + experiment  competition + mass + wing aspect ratio + experiment  competition + mass + burst + wing length + experiment  competition + mass + wing length + wing aspect ratio + experiment  competition + mass + burst + wing aspect ratio + experiment  competition + mass + burst + wing length + wing aspect ratio + experiment | 3  8  8  8  9  9  9  10 | 121.72  130.13  130.85  131.23  132.44  133.01  133.75  135.43 | 0  8.41  9.13  9.51  10.72  11.29  12.03  13.71 | 0.96  0.01  0.01  0.01  0  0  0  0 |
| *AccVD_max_* | 1  2  3  4  5  6  7  8 | **no fixed effects**  competition + mass + wing length + experiment  competition + mass + wing aspect ratio + experiment  competition + mass + burst + experiment  competition + mass + wing length + wing aspect ratio + experiment  competition + mass + burst + wing length + experiment  competition + mass + burst + wing aspect ratio + experiment  competition + mass + burst + wing length + wing aspect ratio + experiment | 3  8  8  8  9  9  9  10 | 111.42  116.51  118.40  118.86  119.05  119.40  121.33  122.10 | 0  5.09  6.98  7.44  7.63  7.98  9.91  10.68 | 0.84  0.07  0.03  0.02  0.02  0.02  0.01  0 |
| *PitchU_vel, avg_* | 1  2  3  4  5  6  7  8 | **competition + mass + burst + experiment**  no fixed effects  competition + mass + burst + wing aspect ratio + experiment  competition + mass + burst + wing length + experiment  competition + mass + wing aspect ratio + experiment  competition + mass + wing length + experiment  competition + mass + burst + wing length + wing aspect ratio + experiment  competition + mass + wing length + wing aspect ratio + experiment | 8  3  9  9  8  8  10  9 | -90.05  -87.57  -87.28  -87.21  -87.10  -86.99  -84.30  -84.18 | 0  2.48  2.77  2.85  2.96  3.07  5.76  5.87 | 0.43  0.12  0.11  0.10  0.10  0.09  0.02  0.02 |
| *PitchD_vel, avg_* | 1  2  3  4  5  6  7  8 | **competition + mass + burst + experiment**  **competition + mass + wing length + experiment**  **competition + mass + burst + wing length + experiment**  **competition + mass + wing aspect ratio + experiment**  **competition + mass + burst + wing aspect ratio + experiment**  competition + mass + wing length + wing aspect ratio + experiment  competition + mass + burst + wing length + wing aspect ratio + experiment  no fixed effects | 8  8  9  8  9  9  10  3 | -80.91  -79.82  -79.47  -79.12  -79.08  -77.83  -77.50  -72.02 | 0  1.09  1.44  1.79  1.83  3.09  3.41  8.89 | 0.30  0.18  0.15  0.12  0.12  0.07  0.06  0 |
| *Yaw_vel, avg_* | 1  2  3  4  5  6  7  8 | **no fixed effects**  competition + mass + burst + experiment  competition + mass + wing length + experiment  competition + mass + wing aspect ratio + experiment  competition + mass + burst + wing length + experiment  competition + mass + burst + wing aspect ratio + experiment  competition + mass + wing length + wing aspect ratio + experiment  competition + mass + burst + wing length + wing aspect ratio + experiment | 3  8  8  8  9  9  9  10 | -73.74  -70.36  -69.88  -68.99  -68.20  -67.43  -66.95  -65.13 | 0  3.38  3.86  4.75  5.54  6.31  6.79  8.61 | 0.64  0.12  0.09  0.06  0.04  0.03  0.02  0.01 |
| *PRT_deg_* | 1  2  3  4  5  6  7  8 | **no fixed effects**  competition + mass + burst + experiment  competition + mass + wing aspect ratio + experiment  competition + mass + wing length + experiment  competition + mass + burst + wing aspect ratio + experiment  competition + mass + burst + wing length + experiment  competition + mass + wing length + wing aspect ratio + experiment  competition + mass + burst + wing length + wing aspect ratio + experiment | 3  8  8  8  9  9  9  10 | 404.24  406.85  406.92  406.99  409.82  409.85  409.93  412.98 | 0  2.61  2.68  2.75  5.58  5.61  5.69  8.74 | 0.51  0.14  0.13  0.13  0.03  0.03  0.03  0.01 |
| *PRT_time_* | 1  2  3  4  5  6  7  8 | **competition + mass + burst + experiment**  **competition + mass + burst + wing aspect ratio + experiment**  **competition + mass + burst + wing length + experiment**  competition + mass + burst + wing length + wing aspect ratio + experiment  competition + mass + wing length + experiment  competition + mass + wing aspect ratio + experiment  competition + mass + wing length + wing aspect ratio + experiment  no fixed effects | 8  9  9  10  8  8  9  3 | -204.98  -203.19  -203.02  -201.08  -200.39  -200.14  -198.27  -190.31 | 0  1.79  1.96  3.90  4.59  4.84  6.71  14.66 | 0.46  0.19  0.17  0.07  0.05  0.04  0.02  0 |
| *Arc_rad_* | 1  2  3  4  5  6  7  8 | **competition + mass + burst + experiment**  **competition + mass + burst + wing aspect ratio + experiment**  competition + mass + burst + wing length + experiment  competition + mass + burst + wing length + wing aspect ratio + experiment  competition + mass + wing aspect ratio + experiment  competition + mass + wing length + experiment  competition + mass + wing length + wing aspect ratio + experiment  no fixed effects | 8  9  9  10  8  8  9  3 | -76.08  -75.59  -73.15  -72.51  -70.43  -68.85  -67.54  -64.94 | 0  0.50  2.93  3.57  5.65  7.23  8.55  11.15 | 0.44  0.34  0.10  0.07  0.03  0.01  0.01  0 |
| *Arc_vel, avg_* | 1  2  3  4  5  6  7  8 | **competition + mass + burst + experiment + days**  competition + mass + burst + wing length + experiment + days  competition + mass + burst + wing aspect ratio + experiment + days  competition + mass + burst + wing length + wing aspect ratio + experiment + days  competition + mass + wing aspect ratio + experiment + days  competition + mass + wing length + experiment + days  competition + mass + wing length + wing aspect ratio + experiment + days  no fixed effects | 9  10  10  11  9  9  10  3 | -18.36  -15.31  -15.28  -12.08  -7.87  -7.76  -4.80  16.13 | 0  3.05  3.07  6.28  10.49  10.60  13.55  34.49 | 0.67  0.15  0.14  0.03  0  0  0  0 |
| *Arc_cent, max_* | 1  2  3  4  5  6  7  8 | **competition + mass + wing aspect ratio + experiment + days**  competition + mass + burst + wing aspect ratio + experiment + days  competition + mass + wing length + wing aspect ratio + experiment + days  competition + mass + burst + wing length + wing aspect ratio + experiment + days  competition + mass + burst + experiment + days  competition + mass + wing length + experiment + days  competition + mass + burst + wing length + experiment + days  no fixed effects | 9  10  10  11  9  9  10  3 | 186.23  188.26  189.03  191.26  192.62  192.71  195.50  213.47 | 0  2.03  2.80  5.03  6.39  6.48  9.27  27.24 | 0.56  0.20  0.14  0.05  0.02  0.02  0.01  0 |
| *PRT%* | 1  2  3  4  5  6  7  8 | **competition + mass + burst + wing length + wing aspect ratio + experiment**  **competition + mass + burst + wing aspect ratio + experiment**  competition + mass + wing aspect ratio + experiment  competition + mass + wing length + wing aspect ratio + experiment  competition + mass + burst + experiment  competition + mass + burst + wing length + experiment  competition + mass + wing length + experiment  no fixed effects | 10  9  8  9  8  9  8  3 | -76.14  -75.27  -73.82  -73.41  -66.99  -65.97  -65.63  -50.33 | 0  0.87  2.32  2.74  9.16  10.17  10.51  25.81 | 0.45  0.29  0.14  0.11  0  0  0  0 |

**1B.** Unstandardized partial regression coefficients and confidence intervals for supported models of maneuvering performance. This table repeats the information in table 5 of the main text, except that it provides unstandardized regression coefficients instead of the standardized beta coefficient. These unstandardized regression coefficients can be interpreted as the change in the response variable per one unit change in continuous predictor variables (i.e., body mass, wing length, wing aspect ratio, days post-capture). Note that burst performance is expressed as residual performance independent of wing morphology, and is therefore unitless.

| model | support for | fixed effects | coef [95% CI] | relative importance | R^2^_GLMM(m)_  burst + morphology + competitor |
| --- | --- | --- | --- | --- | --- |
| *Vel*_max_ | burst | competitor presence  mass  burst  wing length  wing aspect ratio  experiment (CA1)  experiment (CA2)  days post-capture | –0.04 [–0.18, 0.10]  0.27 [–0.03, 0.56]  0.09 [0.001, 0.18]  –0.03 [–0.09, 0.02]  0.28 [–0.19, 0.74]  1.01 [0.59, 1.42]  1.06 [0.68, 1.43]  –0.002 [–0.006, 0.003] | --  --  1.00  0.25  0.26  --  --  -- | 0.28 |
| *AccHor*_max_ | burst + competition | competitor presence  mass  burst  experiment(CA1)  experiment(CA2)  days post-capture | –0.46 [–0.82, –0.11]  0.53 [–0.72, 1.77]  0.39 [0.004, 0.77]  4.01 [2.46, 5.56]  3.68 [2.72, 4.64]  ­–0.01 [–0.03 0.01] | --  --  1.00  --  --  -- | 0.18 |
| *DecHor*_max_ | burst + competition | competitor presence  mass  burst  experiment(CA1)  experiment(CA2)  days post-capture | –0.47 [–0.78, –0.16]  0.79 [–0.34, 1.92]  0.41 [0.06, 0.76]  3.86 [2.47, 5.25]  3.64 [2.76, 4.51]  ­–0.01 [–0.02, 0.01] | --  --  1.00  --  --  -- | 0.19 |
| *AccVU*_max_ | intercept-only | NA | NA | NA | 0 (NA) |
| *AccVD*_max_ | intercept-only | NA | NA | NA | 0 (NA) |
| *PitchU*_vel, avg_ | burst | competitor presence  mass  burst  experiment(CA1)  experiment(CA2) | 0.02 [–0.02, 0.06]  0.004 [–0.10, 0.11]  0.03 [–0.01, 0.07]  0.14 [0.06, 0.23]  0.13 [0.03, 0.23] | --  --  1.00  --  -- | 0.10 |
| *PitchD*_vel, avg_ | competition  + burst | competitor presence  mass  burst  wing length  wing aspect ratio  experiment(CA1)  experiment(CA2) | 0.06 [0.01, 0.10]  ­0.01 [–0.11, 0.14]  0.03 [–0.01, 0.08]  0.02 [–0.01, 0.05]  –0.11 [–0.35, 0.13]  0.19 [0.03, 0.34]  0.22 [0.03, 0.41] | --  --  0.66  0.37  0.28  --  -- | 0.18 |
| *Yaw*_vel, avg_ | intercept-only | NA | NA | NA | 0 (NA) |
| *PRT*_deg_ | intercept-only | NA | NA | NA | 0 (NA) |
| *PRT*_time_ | burst | competitor presence  mass  burst  wing length  wing aspect ratio  experiment(CA1)  experiment(CA2) | –0.001 [–0.01, 0.01]  ­–0.03 [–0.07, 0.01]  –0.02 [–0.03, –0.002]  ­–0.004 [–0.01, 0.01]  0.03 [–0.04, 0.11]  –0.08 [–0.12, –0.04]  –0.11 [–0.16, –0.05] | –  --  1.00  0.21  0.23  –  -- | 0.29 |
| *Arc*_rad_ | burst | competitor presence  mass  burst  wing aspect ratio  experiment(CA1)  experiment(CA2) | –0.02 [–0.07, 0.03]  0.04 [–0.08, 0.16]  0.06 [0.01, 0.10]  –0.16 [–0.39, 0.07]  0.25 [0.12, 0.37]  0.29 [0.06, 0.52] | --  --  1.00  0.44  –  -- | 0.22 |
| *Arc*_vel, avg_ | burst | competitor presence  mass  burst  experiment(CA1)  experiment(CA2)  days post-capture | –0.01 [–0.09, 0.08]  0.08 [–0.15, 0.32]  0.11 [0.04, 0.19]  0.89 [0.59, 1.19]  0.74 [0.56, 0.92]  –0.002 [–0.005, 0.002] | –  –  1.00  –  --  -- | 0.18 |
| *Acc*_cent, max_ | wing shape | competitor presence  mass  wing aspect ratio  experiment(CA1)  experiment(CA2)  days post-capture | 0.29 [–0.37, 0.94]  –0.52 [–1.92, 0.88]  2.88 [0.50, 5.25]  5.93 [4.02, 7.84]  0.85 [–1.59, 3.28]  –0.04 [–0.06, –0.02] | --  --  1.00  --  --  -- | 0.36 |
| *PRT%* | wing shape + competition + burst + wing size | competitor presence  mass  burst  wing length  wing aspect ratio  experiment(CA1)  experiment(CA2) | –0.14 [–0.19, –0.09]  –0.003 [–0.12, 0.12]  0.04 [0.001, 0.09]  –0.03 [–0.05, 0.003]  –0.41 [–0.64, –0.18]  0.17 [–0.03, 0.36]  0.44 [0.19, 0.69] | --  --  1.00  0.61  1.00  --  -- | 0.27 |
|  |  |  |  |  |  |
